# Simultaneous ST-Elevation Myocardial Infarction Without Obstructive Coronary Disease and Intracranial Hemorrhage Complicated by Recurrent Stroke in a Patient With Diffuse Thrombosis

**DOI:** 10.7759/cureus.109919

**Published:** 2026-05-30

**Authors:** Spyridon Karkoulias, Alexandros Tsiavos, Nikolaos P Margos, Konstantinos Kostopoulos

**Affiliations:** 1 1st Cardiology Clinic, General Hospital of Nikaia, Piraeus, Athens, GRC

**Keywords:** antiphospholipid antibody syndrome (aps), catastrophic antiphospholipid syndrome (caps), disseminated intravascular coagulation (dic), intracranial hemorrage, lupus anticoagulant, lupus anticoagulant antibodies, non bacterial endocarditis, seronegative antiphospholipid syndrome, st-elevation myocardial infarction (stemi), trousseau’s syndrome

## Abstract

Simultaneous arterial and venous thrombotic events across multiple vascular territories represent a rare and diagnostically challenging clinical scenario, particularly when accompanied by intracranial hemorrhage that limits therapeutic options. We present the case of a 56-year-old male with ST-elevation myocardial infarction (STEMI) caused by a large thrombus in the left anterior descending artery without underlying stenosis. His course was complicated by intracranial hemorrhage, acute kidney injury, thrombotic microangiopathy features, left ventricular thrombus, and recurrent ischemic strokes involving the left middle cerebral artery (MCA), left posterior cerebral artery (PCA), and acute right MCA occlusion.

Extensive evaluation revealed diffuse thrombosis without a clear unifying diagnosis. Antiphospholipid syndrome was suspected; however, serologic confirmation was inconclusive. Malignancy-associated thrombosis was also considered.

This case demonstrates a complex differential diagnosis, and it highlights the limitations of current diagnostic testing. It also underscores the therapeutic dilemma of managing concurrent thrombosis and intracranial hemorrhage. Despite multidisciplinary care, the patient’s course was fatal.

## Introduction

Diffuse thrombosis involving multiple vascular beds is an uncommon but life-threatening clinical presentation. Such cases require rapid evaluation for underlying hypercoagulable states and prompt treatment. Potential etiologies include antiphospholipid syndrome (APS) [1], immunologic disorders, malignancy-associated thrombosis [2], and thrombotic microangiopathies [3,4]. 

Cerebrovascular manifestations are common in antiphospholipid syndrome (APS) and catastrophic APS (CAPS), often presenting as recurrent arterial or venous strokes, sometimes with hemorrhagic transformation [1]. The co-occurrence of subarachnoid hemorrhage and acute ischemic strokes in multiple territories, as seen in this case, poses a particular diagnostic and therapeutic challenge.

Diagnosis may be particularly challenging in critically ill patients, where laboratory testing is often affected by therapies such as anticoagulation [5,6]. Furthermore, management becomes significantly more complex when thrombotic events coexist with intracranial hemorrhage [7]. Treatment strategies may come into conflict, making clinical decision-making particularly challenging. 

We present a case of simultaneous ST-elevation myocardial infarction (STEMI), intracranial hemorrhage, and recurrent stroke in the setting of diffuse thrombosis, illustrating both diagnostic uncertainty and therapeutic complexity. 

## Case presentation

A 56-year-old male presented with acute chest pain and electrocardiographic findings consistent with anterior STEMI. His medical history included a prior myocardial infarction with stenting of the left anterior descending artery two years prior, deep venous thrombosis of both upper and lower extremities approximately a month prior, chronic kidney disease (CKD), type 2 diabetes mellitus, dyslipidemia, and smoking. The patient's ECG on admission is demonstrated in Figure 1.

**Figure 1:**
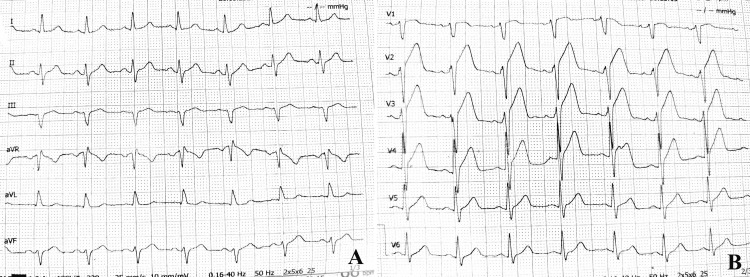
Patient's ECG on Admission Limb leads are shown in panel A. Precordial leads are shown in panel B.

Emergency coronary angiography revealed a large thrombus in the left anterior descending (LAD) artery without underlying stenosis. Balloon angioplasty (Plain Old Balloon Angioplasty (POBA)) was performed, and a flow grade of TIMI II was achieved. However, no stent was placed due to the absence of a culprit stenotic lesion. He was administered loading doses of aspirin (300 mg) and clopidogrel (600 mg). Figure 2 shows the most important aspects of the angiography, and the final result is shown in a short clip (Video 1).

**Figure 2:**
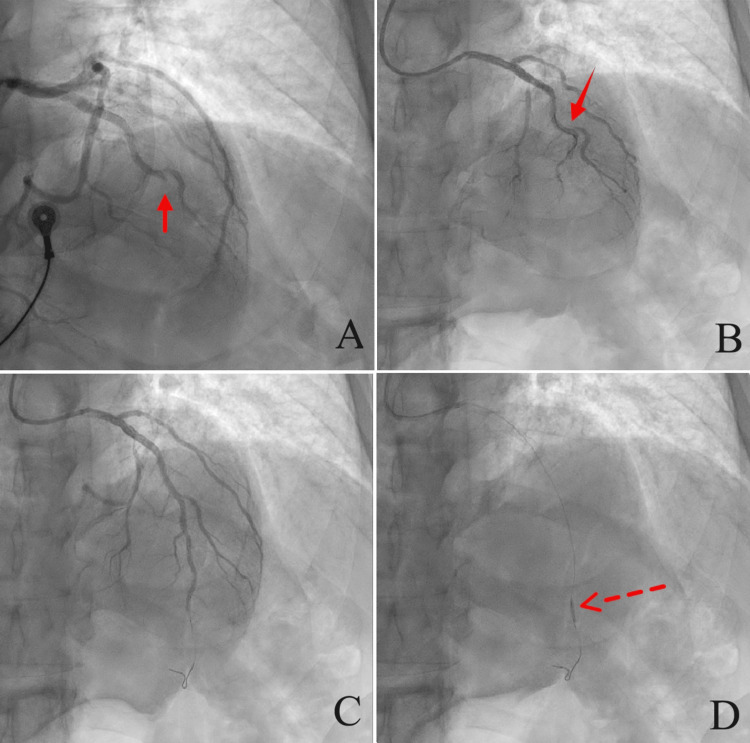
Coronary Angiogram of the Patient In Panel A, the blockage of the LAD is pointed out by the arrow. A significant filling defect can be noted. In Panel B, the guidewire is inserted through the blockage, and there is no underlying stenosis. Panel C depicts further distal advancement of the guidewire, and Panel D illustrates the POBA technique. LAD: left anterior descending artery; POBA: Plain Old Balloon Angioplasty

**Video 1:** Coronary Angiography Final Result

After a few hours, the patient developed a decreased level of consciousness (Glasgow Coma Scale (GCS) 12/15, no focal deficit at that time). Non-contrast brain CT demonstrated mild hyperdensity in the subarachnoid space involving the left frontal and bilateral parieto-occipital regions, consistent with subarachnoid hemorrhage (SAH) of limited extent. No mass effect or hydrocephalus was present. This intracranial hemorrhage, despite being mild, led to discontinuation of antiplatelet and antithrombotic therapy. 

Over the following two days, he was closely monitored in the cardiac ICU, where he retained a left ventricular ejection fraction (LVEF) of approximately 40%. His mental status improved, and after neurosurgical consultation, another head CT was performed, but this time with intravenous (IV) contrast. Repeat non-contrast and CT angiography (CTA) of the brain showed stable to slightly decreased hyperdensity of the SAH (no expansion). The hemorrhage was judged to be in the early absorptive phase, and neurosurgical consultation deemed it safe to cautiously restart antithrombotic therapy. Given the risk of hemorrhage recurrence, anticoagulation was cautiously resumed with a combination of low molecular weight heparin (LMWH, enoxaparin 0.4 mg/kg) once daily due to his chronic kidney failure and acetylsalicylic acid (ASA - aspirin 100 mg daily). 

Over the following days, the patient developed acute kidney injury superimposed on his chronic kidney failure. The administration of IV contrast during his last head CT and during the angiography probably played a role in this occurrence. Furthermore, he developed mild thrombocytopenia (from 155,000/μL to 125,000/μL) and anemia. Peripheral blood smear demonstrated schistocytes, raising concern for thrombotic microangiopathy. There were limitations in this report because the degree and amount of schistocytes were not described, and no haptoglobin was measured. Moreover, bilirubin was measured a few days prior and was normal but was not repeated afterwards. It is known that lactate dehydrogenase (LDH) was measured at approximately 750 IU/L (normal range 80-235 IU/L). Reticulocytes were found to be close to upper limits (approximately 4K/μL). Vitamin Β12 was normal, iron (Fe) was 20 μg/dl, ferritin (FERR) was 1108 ng/ml, folate was 2. 87 ng/ml, and transferrin (TRF) was 154 mg/dl. Heparin-induced thrombocytopenia (HIT) testing was negative. Moreover, coagulation parameters, including activated partial thromboplastin time and fibrinogen levels, were only mildly abnormal and not consistent with disseminated intravascular coagulation (DIC). 

Extensive thrombophilia and autoimmune workup was notable for a heterozygous prothrombin G20210A mutation and a borderline antinuclear antibody (ANA) result. Homocysteine levels were within normal limits. A borderline lupus anticoagulant was also noted in a recent test the patient had undergone a week prior; however, this test had been performed under recent rivaroxaban therapy, limiting its interpretability. Finally, cryoglobulins were negative, and complement testing revealed a slightly elevated C3 level (188 mg/dL) consistent with an acute phase response, while C4 was within normal limits. Tables 1, 2 show the results of the autoimmune and thrombophilia testing.

**Table 1:** Autoimmune and Thrombophilia Panel ELISA: enzyme-linked immunosorbent assay; IFA: indirect fluorescent antibody (assay); M2EP: mitochondrial antigen M2 epitope; ANA: antinuclear antibody; F-ANA: fluorescent antinuclear antibody; ENA: extractable nuclear antigen; AMA: antimitochondrial antibody; ds-DNA-NCX: double-stranded DNA (Crithidia luciliae); P-ANCA: perinuclear antineutrophil cytoplasmic antibody; C-ANCA: cytoplasmic antineutrophil cytoplasmic antibody; MPO: myeloperoxidase; PR3: proteinase 3; GBM: glomerular basement membrane

Test	Result	Reference Range
M2EP (ELISA)	2.68 (Negative)	<25 U/mL
ANA	Weakly positive 1:160	<1:160
F-ANA	Fine speckled	Not Applicable
ENA Antibodies (ELISA)	0.50 (Negative)	<1.40
Anti-smooth muscle antibody (IFA)	Negative	<1:40
AMA (IFA)	Negative	<1:40
Anti-cardiolipin IgG (ELISA)	11.60 (Negative)	<12 GPL (negative), 12-18 (borderline), >18 (positive)
Anti-cardiolipin IgM (ELISA)	3.37 (Negative)	<12 MPL (negative), 12-18 (borderline), >18 (positive)
ds-DNA-NCX (ELISA)	2.18 (Negative)	Positive >100 U/mL
Anti-β2-glycoprotein I IgM (ELISA)	1.21 (Negative)	<12 U/mL (negative), 12-18 (borderline), >18 (positive)
Anti-β2-glycoprotein I IgG (ELISA)	4.89 (Negative)	<12 U/mL (negative), 12-18 (borderline), >18 (positive)
Anti-phosphatidylserine IgM	Negative	<10 U/mL
Anti-phosphatidylserine IgG	Negative	<10 U/mL
P-ANCA (IFA)	Negative	<1:20
C-ANCA (IFA)	Negative	<1:20
Anti-MPO (ELISA)	0.62 (Negative)	<12 U/mL (negative), 12-18 (borderline), >18 (positive)
Anti-PR3 (ELISA)	1.04 (Negative)	<12 U/mL (negative), 12-18 (borderline), >18 (positive)
Anti-GBM (IFA)	Negative	<1:10
Anti-GBM (ELISA)	1.66 (Negative)	<20 U/mL

**Table 2:** Immunoglobulin and Complement Levels

Test	Result	Reference Range
IgG	1360.00 mg/dL	700 - 1600 mg/dL
IgA	356.00 mg/dL	70 - 400 mg/dL
IgM	53.50 mg/dL	40 - 230 mg/dL
C3 Complement	188.00 mg/dL	90 - 180 mg/dL
C4 Complement	36.90 mg/dL	10 - 40 mg/dL

Subsequently, the patient developed mild mental status changes without any focal motor deficit. GCS was 13/15 (E3, V4, M6). The National Institutes of Health Stroke Scale (NIHSS) score was estimated to be 4 (primarily confusion and mild dysphasia). Non-contrast CT brain revealed a large hypodense area (90×40 mm) in the left parietal and occipital lobes, consistent with an acute ischemic stroke in the left middle cerebral artery (MCA) territory. Alberta Stroke Program Early CT Score (ASPECTS), based on the extent of hypodensity on CT, corresponded to an approximate score of 7. Because the patient had no muscle weakness, symptoms were mild, and the CT was performed many hours after onset, it was decided to manage medically and close monitoring rather than pursue thrombolysis or thrombectomy. Intravenous thrombolysis was also contraindicated due to the recent subarachnoid hemorrhage.

Transthoracic echocardiography revealed a reduced LVEF of approximately 40%, an apical left ventricular thrombus, and mitral valve vegetations suggestive of endocarditis. Mitral regurgitation was also present. The presence of apical thrombus was also confirmed with the use of contrast. However, blood cultures came back negative. These findings were suggestive of either non-bacterial thrombotic endocarditis or Libman-Sacks endocarditis. It is worth noting that throughout his hospitalization, the patient retained high white blood cell counts (>20 K/μL) and C-reactive protein levels (>200 mg/L with normal range <5 mg/L) but very low procalcitonin levels (normal). He received empiric antibiotic treatment with minimal change. The following videos (Videos 2-5) demonstrate the key findings described during the transthoracic cardiac ultrasound.

**Video 2:** Transthoracic Echocardiogram - Apical Four-Chamber View This clip demonstrates the reduced left ventricular ejection fraction (LVEF). The evaluation of the apex significantly raises suspicion for an apical thrombus. Note also the mitral valve vegetations suggestive of non-bacterial thrombotic endocarditis or Libman-Sacks endocarditis.

**Video 3:** Transthoracic Echocardiogram - Color Doppler in Apical Four-Chamber View This video demonstrates the presence of mitral regurgitation. The characteristics of the jet suggest at least moderate severity.

**Video 4:** Transthoracic Echocardiogram - Apical Three-Chamber View The apical thrombus is more clearly appreciated in this plane.

**Video 5:** Mitral Valve - Zoomed Three-Chamber View Close-up zoom view of the mitral valve in the three-chamber plane, clearly depicting the irregular vegetation.

However, the following day, he acutely deteriorated to GCS 7 (E1, V2, M4) with new right-sided hemiplegia. NIHSS was 22. Emergency non-contrast CT and CTA were performed. The CT showed: (i) a new, likely acute, small hypodense area in the right occipital lobe (right posterior cerebral artery - PCA - territory), (ii) the previously noted large infarct in the left parietal and occipital lobes (left MCA territory) as seen in Figure 3, and (iii) a new left occipital-thalamic infarct (left PCA territory). CTA revealed occlusion of the right internal carotid artery (C7 segment) and the entire right MCA (M1 segment and branches), as well as occlusion of the peripheral left posterior cerebral artery (PCA). The patient was intubated due to declining GCS. Chest CT was also performed. It showed a well-placed endotracheal tube, mild lymphadenopathy, and no other major findings.

**Figure 3:**
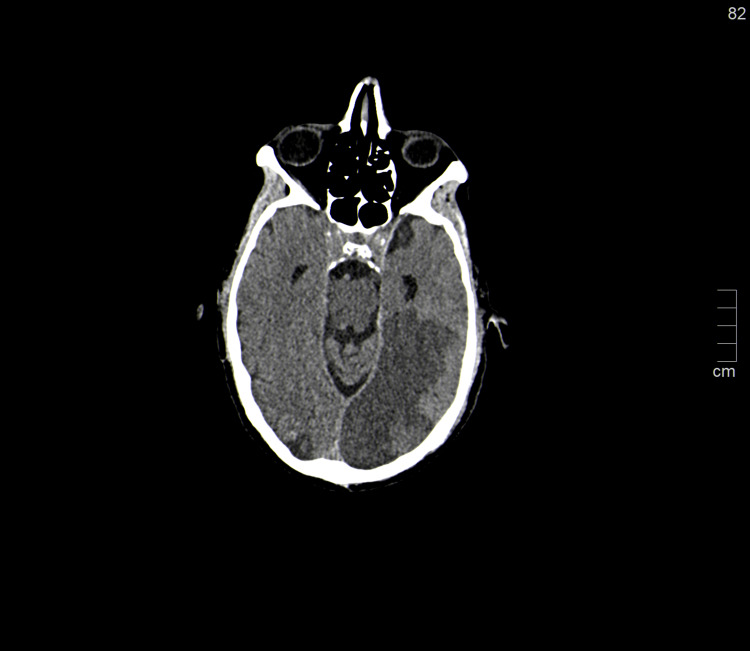
Non-contrast CT Demonstrating an Extensive Hypodense Area in the Left Hemisphere Consistent With the Known Left MCA Territory Ischemic Infarct.

The patient was transferred to a tertiary center for thrombectomy. Mechanical thrombectomy was performed with groin puncture approximately 5-6 hours after symptom onset (due to transfer delay). The Thrombolysis in Cerebral Infarction (TICI) grade was 2B after two passes. During the procedure, multiple thrombi were noted in the femoral artery and throughout the vascular system, as was mentioned after communication with the physicians involved in the procedure.

The presence of rapidly progressive thrombosis involving multiple vascular territories within a short time frame implied that this systemic prothrombotic state was of significant intensity and had to be addressed more aggressively. It was decided to increase LMWH twice daily despite his CKD and to administer corticosteroids. It was also suggested that a CT scan of the abdomen should be performed at a later time to evaluate for occult malignancy.

Unfortunately, the course of the patient's illness allowed time for only limited administration of corticosteroids and did not allow for the titration of the LMWH. Post-procedure CT revealed a significant intraparenchymal hemorrhage associated with neurological deterioration. The exact European Cooperative Acute Stroke Study (ECASS) grade was not documented in the available radiology report. He deteriorated sharply following the hemorrhagic transformation, and ultimately passed away in less than 24 hours after the thrombectomy procedure.

## Discussion

This case represents a diagnostically and therapeutically complex scenario involving ST-elevation myocardial infarction without obstructive coronary disease, intracranial hemorrhage, acute kidney injury, features of thrombotic microangiopathy, and recurrent ischemic strokes occurring within a short timeframe in a patient with recent deep venous thrombosis. Despite extensive evaluation, no definitive unifying diagnosis was established. The discussion addresses the differential diagnosis of the thrombotic process, the hematological findings, valvular involvement, systemic inflammation, and the associated therapeutic challenges. 

Significance of thrombosis without obstructive coronary disease

The presence of a high thrombus burden in the left anterior descending artery without an underlying stenotic lesion is a key diagnostic finding. In patients with prior coronary artery disease, recurrent events are often attributed to plaque progression or in-stent thrombosis. However, the absence of a culprit lesion suggests an alternative mechanism, most likely a systemic prothrombotic state. This interpretation is supported by the patient’s recent history of deep venous thrombosis involving both upper and lower extremities, an atypical distribution that raises suspicion for an underlying hypercoagulable condition. Together, these findings indicate that conventional cardiovascular risk factors alone cannot explain the clinical presentation. 

Hematological findings and thrombotic microangiopathy 

The presence of schistocytes raised concern for thrombotic microangiopathy (TMA). The differential diagnosis includes thrombotic thrombocytopenic purpura (TTP), atypical hemolytic uremic syndrome (aHUS), disseminated intravascular coagulation (DIC), antiphospholipid syndrome-associated TMA, and malignancy-related microangiopathy [3]. 

The platelet count declined from 155,000/μL to approximately 125,000/μL, representing mild rather than severe thrombocytopenia. This makes TTP and aHUS less likely in this setting, as these conditions typically present with more profound thrombocytopenia [3]. ADAMTS13 activity was not measured, which represents a limitation, but the clinical picture was not strongly suggestive of severe deficiency. 

Coagulation parameters, including activated partial thromboplastin time and fibrinogen levels, remained near normal. These findings make DIC less likely, which typically presents with coagulopathy and hypofibrinogenemia. The absence of DIC also reduces the likelihood of malignancy-related microangiopathy as the primary mechanism. 

Overall, the hematological findings represent features most consistent with a TMA spectrum not clearly attributable to DIC or complement-mediated disease [4]. 

Differential diagnosis of the underlying hypercoagulable state 

*Antiphospholipid Syndrome and Catastrophic APS* 

Antiphospholipid syndrome (APS) is an acquired thrombophilia defined by the presence of antiphospholipid antibodies in association with thrombosis [1]. According to the updated Sydney classification criteria, a definite APS diagnosis requires at least one clinical event (thrombosis) and persistently positive antiphospholipid antibodies (lupus anticoagulant, anticardiolipin, or anti‑β2 glycoprotein I) on two or more occasions at least 12 weeks apart. Its most severe form, catastrophic APS (CAPS), involves rapid multiorgan thrombosis (three or more organs) within a short period (<1 week). Histopathological confirmation of small‑vessel occlusion is required to make a definitive diagnosis [4,8]. 

The clinical presentation in this case - multiorgan involvement, rapid progression, and TMA features - is suggestive of CAPS [8,9]. However, diagnostic confirmation requires persistent antiphospholipid antibody positivity on two or more occasions at least 12 weeks apart (Sydney criteria). In this patient, anticardiolipin antibodies were negative, anti-β2 glycoprotein I antibodies were negative, and lupus anticoagulant was borderline.

Importantly, lupus anticoagulant testing had been performed while the patient was on rivaroxaban, which is known to produce false-positive results [5,6]. Therefore, this finding cannot be considered reliable. Moreover, the patient died before repeat testing at 12 weeks could be performed. Without confirmed serological evidence, APS and CAPS cannot be definitively diagnosed. 

Seronegative CAPS has been described but remains a diagnosis of exclusion [10]. Furthermore, definite CAPS also requires histopathological confirmation of small‑vessel occlusion, which was not available (no autopsy). In the absence of histopathological confirmation, this case is best described as clinically suggestive but not diagnostic of CAPS. 

*Trousseau Syndrome* 

Malignancy-associated thrombosis was also considered [2]. The presence of thrombosis in multiple vascular territories and valvular vegetations raises this possibility. However, thoracic imaging did not identify a clear malignancy, and abdominal imaging was not performed, leaving a diagnostic gap. Additionally, the absence of DIC makes malignancy-associated microangiopathy less likely. While malignancy cannot be excluded, it is not strongly supported by the available data.

Hereditary Thrombophilia 

The patient’s heterozygous prothrombin G20210A mutation likely contributed to thrombotic risk but does not explain the severity or distribution of thrombosis [11]. It is best considered a contributing factor within a multifactorial process. 

*Valvular Lesions* 

Mitral valve vegetations raised the differential of infective endocarditis, non-bacterial thrombotic endocarditis (NBTE), and Libman-Sacks endocarditis. 

Negative blood cultures and the absence of clear infectious features make infective endocarditis less likely [12]. In the context of suspected APS, Libman-Sacks endocarditis is a more probable explanation [13]. NBTE remains a consideration, particularly in the context of possible malignancy [14]. 

These lesions represent a potential embolic source and may have contributed to the recurrent strokes [13,15]. 

Cardioembolic Mechanism 

The identification of an apical left ventricular thrombus provides a clear mechanism for recurrent embolic strokes. This is a known complication of anterior STEMI with reduced ejection fraction and regional wall motion abnormalities [16]. 

However, this finding alone does not explain the systemic distribution of thrombosis, further supporting the presence of an underlying prothrombotic state. 

*Systemic Inflammation* 

The patient exhibited persistently elevated inflammatory markers without an identifiable infectious source. This suggests a systemic inflammatory response. 

In CAPS, inflammation plays a central role in promoting thrombosis through cytokine-mediated endothelial activation. At the same time, systemic inflammation itself can act as a trigger for CAPS. This bidirectional relationship makes it difficult to determine whether inflammation was a cause or consequence of the thrombotic process in this case [9]. 

Therapeutic dilemmas 

Management was particularly challenging due to the coexistence of thrombosis and intracranial hemorrhage. Antithrombotic therapy was initially discontinued and later cautiously reintroduced following clinical and radiological improvement. 

Anticoagulation decisions were further complicated by acute kidney injury. The subsequent identification of a left ventricular thrombus required escalation of anticoagulation despite ongoing hemorrhagic risk. 

There are no clear guideline recommendations for this clinical scenario. Randomized trials such as SoSTART and RESTART have primarily addressed anticoagulation resumption after intracranial hemorrhage (ICH) for atrial fibrillation or venous thromboembolism, but not for catastrophic APS or similar hypercoagulable states. Management relied on multidisciplinary decision-making and individualized risk assessment [7]. 

*Role of Plasma Exchange* 

In suspected CAPS, treatment typically includes anticoagulation, corticosteroids, and plasma exchange or intravenous immunoglobulin [8,9]. Plasma exchange was not performed in this case due to concern for intracranial hemorrhage, hemodynamic instability, acute kidney injury, and the absence of confirmed antiphospholipid antibody positivity. The procedure was deemed too high-risk given the competing hemorrhagic and hemodynamic concerns. However, seronegative CAPS, while a remote possibility, remains the most unifying diagnosis. 

The example of this case indicates that clinical judgment, in selected cases, may complement strict diagnostic criteria. Such an approach may support earlier treatment initiation in suspected CAPS before clinical deterioration. This case also highlights the fact that the most appropriate treatment for a suspected diagnosis may not be feasible due to competing clinical risks. 

## Conclusions

This case highlights the complexity of diagnosing and managing diffuse thrombosis involving multiple vascular territories. Although antiphospholipid syndrome was suspected, diagnostic confirmation was inconclusive. Specifically, definite catastrophic APS could not be established because the Sydney criteria (repeat antiphospholipid antibody testing after 12 weeks) and histopathological confirmation of small‑vessel occlusion were not fulfilled - the patient died before these could be obtained, and an autopsy was not performed.

This case report illustrates very well some valuable learning points. Physicians should keep in mind that antiphospholipid antibody testing performed during treatment with direct oral anticoagulants may yield unreliable results and should be interpreted with caution. Moreover, early and complete oncological evaluation should be considered in cases of unexplained multivessel thrombosis. In this case, as well as in similar instances, the presence of schistocytes with near-normal coagulation parameters should prompt consideration of immune-mediated TMA rather than DIC. Finally, some cases of catastrophic thrombosis may remain without a definitive diagnosis. Nevertheless, prompt empiric treatment should be initiated based on clinical judgment, even in the absence of diagnostic certainty. Clinicians should be aware of the limitations of thrombophilia testing in acute settings, especially when time is scarce. A close multidisciplinary approach involving cardiology, neurology, nephrology, internal medicine, and hematology teams is essential when significant therapeutic challenges are posed by competing risks of thrombosis and hemorrhage.
